# The importance of the rotor in hydrazone-based molecular switches

**DOI:** 10.3762/bjoc.8.98

**Published:** 2012-06-13

**Authors:** Xin Su, Timo Lessing, Ivan Aprahamian

**Affiliations:** 1 Department of Chemistry, Dartmouth College, Hanover, New Hampshire 03755, United States; 2 Department of Chemistry, Heinrich-Heine-Universität Düsseldorf, Universitätsstr. 1, 40225 Düsseldorf, Germany

**Keywords:** *E*/*Z* isomerization, hydrazone, molecular switches, pH activation, structure–property analysis

## Abstract

The pH-activated *E*/*Z* isomerization of a series of hydrazone-based systems having different functional groups as part of the rotor (R = COMe, CN, Me, H), was studied. The switching efficiency of these systems was compared to that of a hydrazone-based molecular switch (R = COOEt) whose *E*/*Z* isomerization is fully reversible. It was found that the nature of the R group is critical for efficient switching to occur; the R group should be a moderate H-bond acceptor in order to (i) provide enough driving force for the rotor to move upon protonation, and (ii) stabilize the obtained *Z* configuration, to achieve full conversion.

## Findings

Nature is full of elegant examples of perfectly designed biological motors and machines [1] that perform delicate and precise tasks. Primitive as they may be, numerous artificial molecular machines [2–6] have been developed that strive to mimic their biological counterparts as far as function is concerned. As part of these efforts, a variety of molecular systems have been developed that can perform different types of motion (e.g*.*, translation, rotation) in response to chemical [7–9], electrochemical [10–13], and photochemical stimuli [14–18]. One of the benefits of artificial molecular switches and machines is that their output can be controlled or fine-tuned by altering their components [19–21]. A relevant example in this context is Feringa’s overcrowded alkene-based light-driven rotary switches that can be induced to rotate at different rates by replacing a naphthyl group in the upper-half of the molecule (i.e., the rotor) with a less sterically hindered benzothiophenyl group [20].

**Scheme 1:**
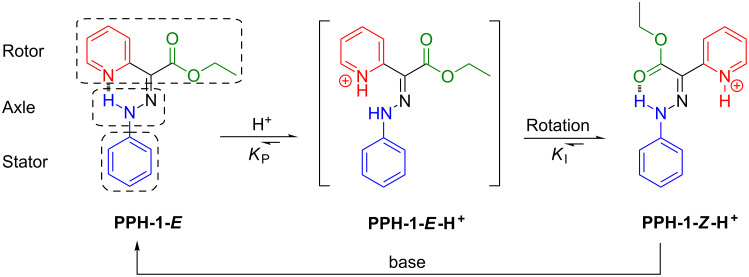
The acid-activated switching process of **PPH-1**.

Previously, we have shown that hydrazone-based rotary switches can change their configuration (i.e., *E*/*Z* isomerization) as a function of pH [22–24], or upon the addition of a Lewis acid (i.e., Zn^2+^) [25]. The simplest hydrazone switch (**PPH-1**, Scheme 1) for example, exists mainly as its *E* isomer (**PPH-1-*E***) in solution, as illustrated by the *E*/*Z* isomer ratio of 93:7 in CD_3_CN. Protonation of **PPH-1-*E*** with acid results in an intermediate **PPH-1-*E*-H^+^**, which quickly isomerizes to **PPH-1-*Z*-H^+^**, which is the more stable isomer. During this process, an *E*/*Z* isomerization takes place, which can be fully reversed by the addition of base to the solution.

**Scheme 2:**
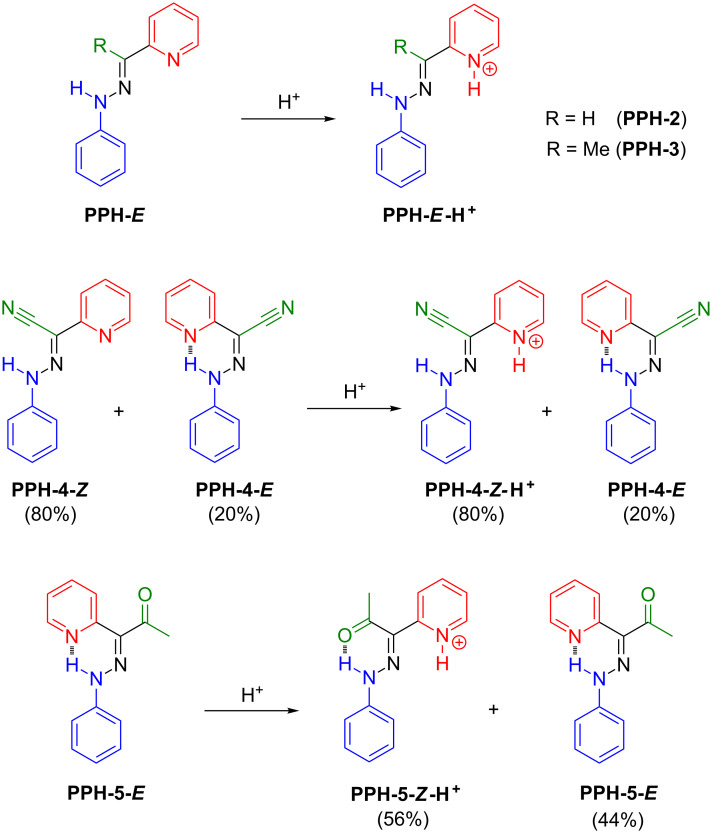
The hydrazone-based molecular systems that were analysed in this paper, each having different rotors. The stable isomer(s) in solution and their protonation products are shown.

In order to fine tune the properties of the hydrazone switches, we studied the effect of different R groups in the rotor part (Scheme 2) on the switching cycle. The target hydrazones were synthesized either by the direct condensation of phenylhydrazine with the corresponding aldehyde (**PPH-2**) or ketone (**PPH-3**), or by Japp–Klingemann reaction (**PPH-4**, **PPH-5**) [26]. The NMR spectroscopy and mass spectrometry characterizations show results consistent with the previously reported data [26].

In certain cases it has been shown that intramolecularly H-bonded hydrazones exist predominantly as the kinetically stable *Z* isomer in solution [27–29]. We were expecting that the intramolecular H-bonds in **PPH-2** and **PPH-3** would drive them to adopt the *Z* configuration in solution as well, leading to a low-field-lying NH signal (12–16 ppm) [22–24]. However, this is not the case with **PPH-2**. The hydrazone N–H proton in **PPH-2** resonates at 8.95 ppm, which clearly shows that it is not H-bonded to the pyridyl nitrogen, indicating that the *E* configuration is the predominant isomer in solution (CD_3_CN). The addition of trifluoroacetic acid (TFA) only results in a general downfield shift of the aromatic and the hydrazone N–H proton signals as a result of protonation, which reaches saturation with 3 equiv of TFA. Unlike in **PPH-1**, signals from other species are not observed during the course of protonation, suggesting that the protonation of **PPH-2** with TFA is a fast equilibrium, and that, as expected, it does not cause any isomerization. Similar to **PPH-2**, the ^1^H NMR spectrum of **PPH-3** shows a signal for the hydrazone N–H proton at 8.24 ppm indicating that it too is in the *E* configuration. The protonation of **PPH-3** with TFA is a fast equilibrium as well, without any indication of rotary motion (i.e., isomerization).

On the other hand, **PPH-4**, in which R is a strong electron-withdrawing group (–CN) shows two sets of signals in the ^1^H NMR spectrum (CD_3_CN), indicating that two isomers, having a 4:1 ratio, coexist in solution. The major isomer shows a hydrazone N–H signal at 9.60 ppm, indicating that it is the *Z* isomer, in which an intramolecular H-bond is not present. On the other hand, the hydrazone N–H signal of the minor isomer resonates at 15.12 ppm, which is characteristic of H-bonded N–H signals, suggesting that the minor isomer is actually the *E* configuration. Such an unusual *E*/*Z* isomer ratio was reported before for similar systems, and it was attributed to kinetic stability of the *Z* isomer, in addition to solvent effects [27–29]. The titration of **PPH-4** with TFA only affects the major isomer (*Z*), while the minor isomer (*E*) remains intact even in the presence of 10 equiv of TFA. The changes in the ^1^H NMR spectrum of **PPH-4-*Z*** are similar to those of **PPH-2** and **PPH-3**, except for the fact that it requires an excess of TFA (ca. 10 equiv) for the protonation to reach saturation. This observation can be attributed to the strong electron-withdrawing nature of the CN group, which drastically decreases the basicity of the pyridyl nitrogen. Furthermore, since the pyridyl nitrogen in **PPH-4-*E*** is H-bonded to the hydrazone N–H, the basicity of **PPH-4-*E*** becomes even lower, which explains why **PPH-4-*E*** does not become protonated even in the presence of 10 equiv of TFA.

Structurally, **PPH-5** is the closest to **PPH-1**, that is, instead of an acyl ester group, **PPH-5** has an acetyl residue as the R group. The ^1^H NMR spectrum of **PPH-5** in CD_3_CN shows only one set of signals, and a sharp singlet at 14.54 ppm for the hydrazone N–H proton, indicating that it is H-bonded to the pyridyl nitrogen. Since the acetyl group is a less effective H-bond acceptor than ethyl ester, it is reasonable that **PPH-5** exists exclusively in the *E* form in solution. When TFA is added to the solution, a second set of signals arises, which grows as the amount of acid increases. The protonation of the pyridyl ring results in the downfield shift of the aromatic signals, except for proton H1, which shifts from 8.92 to 8.70 ppm as it is no longer affected by the H-bond [22–24]. Moreover, the hydrazone N–H signal shifts to a higher field (13.22 ppm) in congruence with what is observed in **PPH-1** [24]. These changes are consistent with those observed during the acid-activated switching of **PPH-1**, suggesting that **PPH-5** switches from the *E* to the *Z* configuration upon protonation. However, the switching process of **PPH-5** is relatively inefficient as there is still ca. 44% of **PPH-5-*E*** remaining in solution even when 30 equiv of TFA is added.

In order to rationalize the different behaviour of the structurally similar switches, **PPH-1** and **PPH-5**, a quantitative evaluation of the thermodynamic process is necessary. Taking a look at the acid-activated switching process of **PPH-1** (Scheme 1), we can formulate the following equations for the acid-induced *E*/*Z* isomerization:

[1]
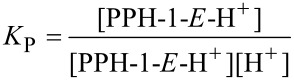


[2]
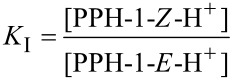


[3]
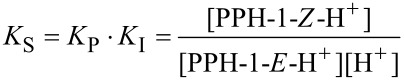


where *K*_P_ is the equilibrium constant of the protonation step, *K*_I_ is the equilibrium constant for the rotation process, and *K*_S_ is the overall equilibrium constant for the switching reaction. The p*K*_a_ of **PPH-1** is actually log_10_*K*_P_, so *K*_S_ also equals 
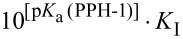
. From the above equations, it becomes clear that *K*_S_ can be used as an index to evaluate the feasibility of the switching process in hydrazone-based switches; the larger the *K*_S_ value, the easier the switching process. In the case of **PPH-1** versus **PPH-5**, since the acetyl group is a stronger electron-withdrawing group than the ester group, the basicity (p*K*_a_) of the pyridyl group in **PPH-1** will be higher than in **PPH-5**. Moreover, the ester group is a better H-bond acceptor than the acetyl group, which means that the protonated *Z* configuration of **PPH-1** is more stable than that of **PPH-5**, resulting in a larger *K*_I_ for **PPH-1**. Thus, it can be qualitatively deduced that **PPH-1** has a larger *K*_S_ than **PPH-5**, suggesting that **PPH-1** is a more ideal system to be used as a molecular switch. This analysis is clearly in line with the acid switching experiments that show that **PPH-1** can be fully switched, whereas **PPH-5** cannot.

## Conclusion

In summary, we have synthesized four hydrazone-based systems having different R groups as part of the rotor section. The role of the R group was assessed *vis-à-vis* the switching of the system, and it was found that for the switch to operate effectively it is crucial that (1) the R group be able to offer a second H-bond-accepting site in order to provide enough driving force for the rotor to move; and (2) the R group be a moderate H-bond acceptor, otherwise the isomer generated will not be stable enough to enable full conversion (isomerization).

## Supporting Information

File 1 Experimental section and acid titration of the hydrazone compounds.
