# Supplementary material for: The importance of the rotor in hydrazone-based molecular switches
Source: Beilstein J Org Chem. 2012 Jun 13;8:872–6. doi: 10.3762/bjoc.8.98 (PMC3388876; doi:10.3762/bjoc.8.98)
Supplement: File 1 — Experimental section and acid titration of the hydrazone compounds. [file Beilstein_J_Org_Chem-08-872-s001.pdf]

Supporting Information

for

The importance of the rotor in hydrazone-based molecular switches

Xin Su[1], Timo Lessing[2] and Ivan Aprahamian*[1]

Address: [1]Department of Chemistry, Dartmouth College, Hanover, New Hampshire 03755, United States and [2]Department of Chemistry, Heinrich-Heine-Universität Düsseldorf, Universitätsstr. 1, 40225 Düsseldorf, Germany

Email: ivan.aprahamian@dartmouth.edu
Homepage: www.dartmouth.edu/~aprahamian

* Corresponding author

Experimental section and acid titration of the hydrazone compounds

Table of Contents

Experimental section.. S3

Acid titration of the hydrazone compounds S4

Experimental section

General: All reagents and starting materials were purchased from Acros, Alfa Aesar and TCI-America, and used without further purification. Column chromatography was performed on silica gel (Silicycle, 230–400 mesh). The melting point was measured on an Electrothermal 9100 instrument in open capillary tubes without thermometer correction. Deuterated solvents were purchased from Cambridge Isotope Laboratories and used as received. NMR spectra were recorded on a 500 MHz spectrometer, with working frequencies of 499.87 MHz for ^{1}H nuclei and 125.7 MHz for ^{13}C nuclei, respectively. Chemical shifts are quoted in ppm relative to tetramethylsilane, using the residual solvent peak as a reference standard.

Acid titration of the hydrazone compounds

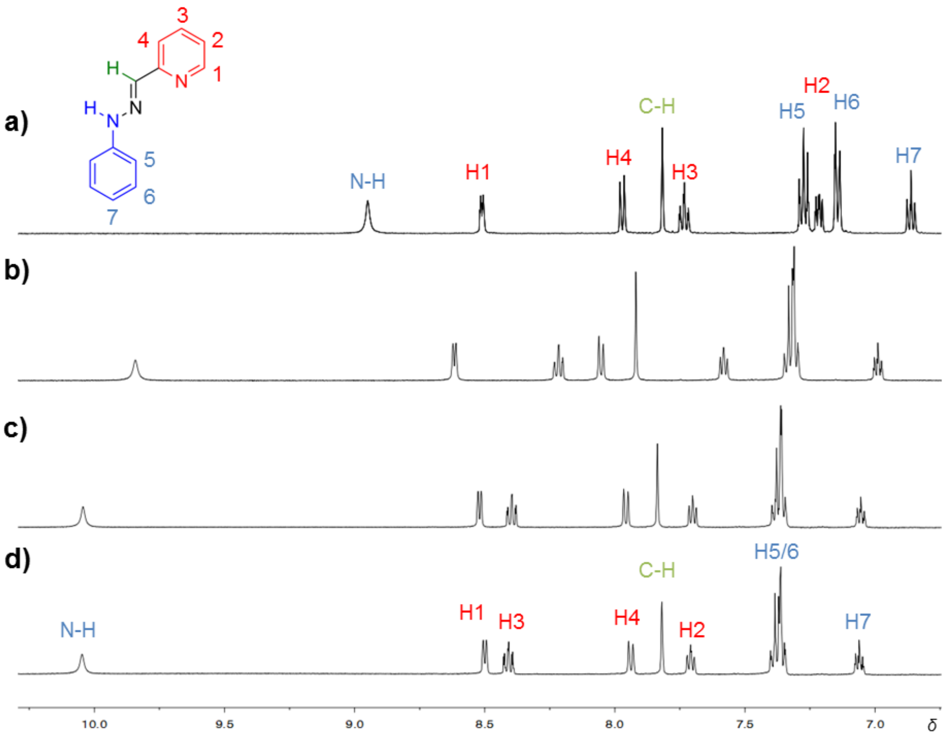

Figure S1: The titration of **PPH-2** with TFA followed by ^{1}H NMR spectroscopy (CD_3CN, 294 K). The **PPH-2** solution (1.0×10^{-2} M) was titrated with 0, 1, 3 and 5 equiv of TFA (**a**–**d** respectively).

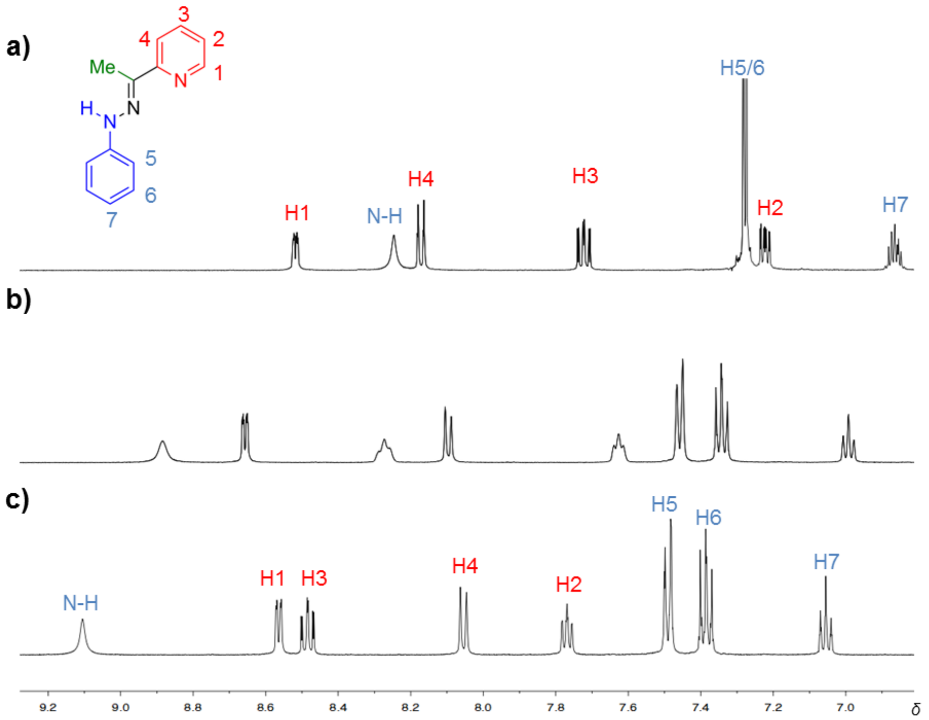

Figure S2: The titration of **PPH-3** with TFA followed by ^{1}H NMR spectroscopy (CD_3CN, 294 K). The **PPH-3** solution (1.0×10^{-2} M) was titrated with 0, 1, and 3 equiv of TFA (**a**–**c** respectively).

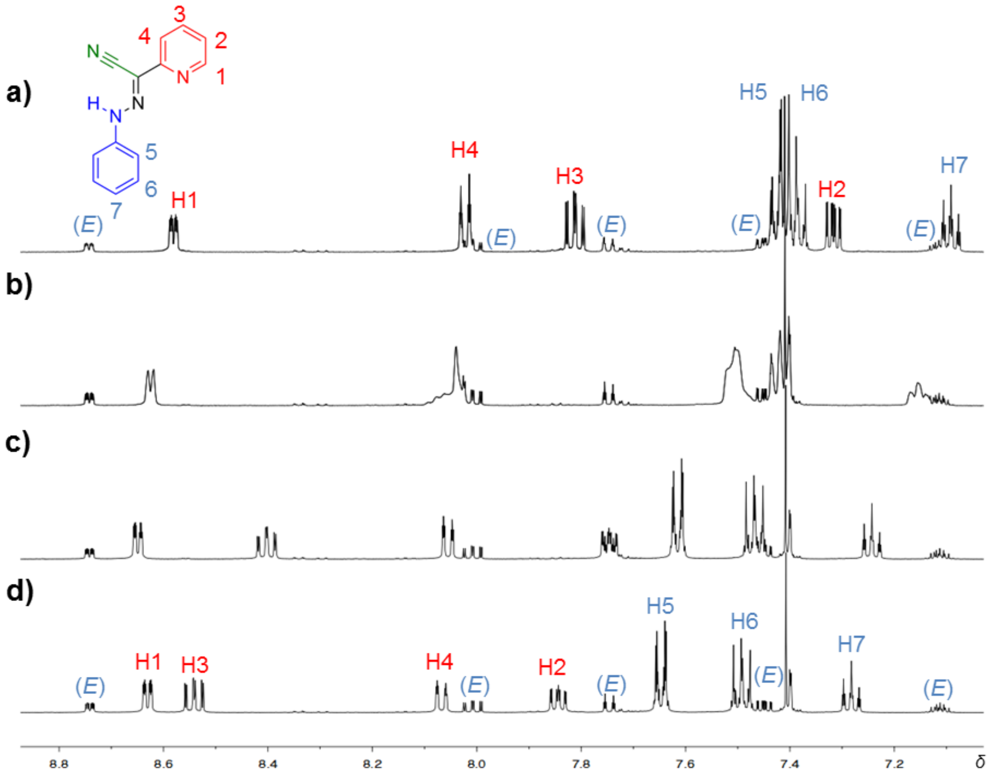

Figure S3: The titration of **PPH-4** with TFA followed by ^{1}H NMR spectroscopy (CD_3CN, 294 K). The **PPH-4** solution (1.0×10^{-2} M) was titrated with 0, 1, 4 and 10 equiv of TFA (**a**–**d** respectively).

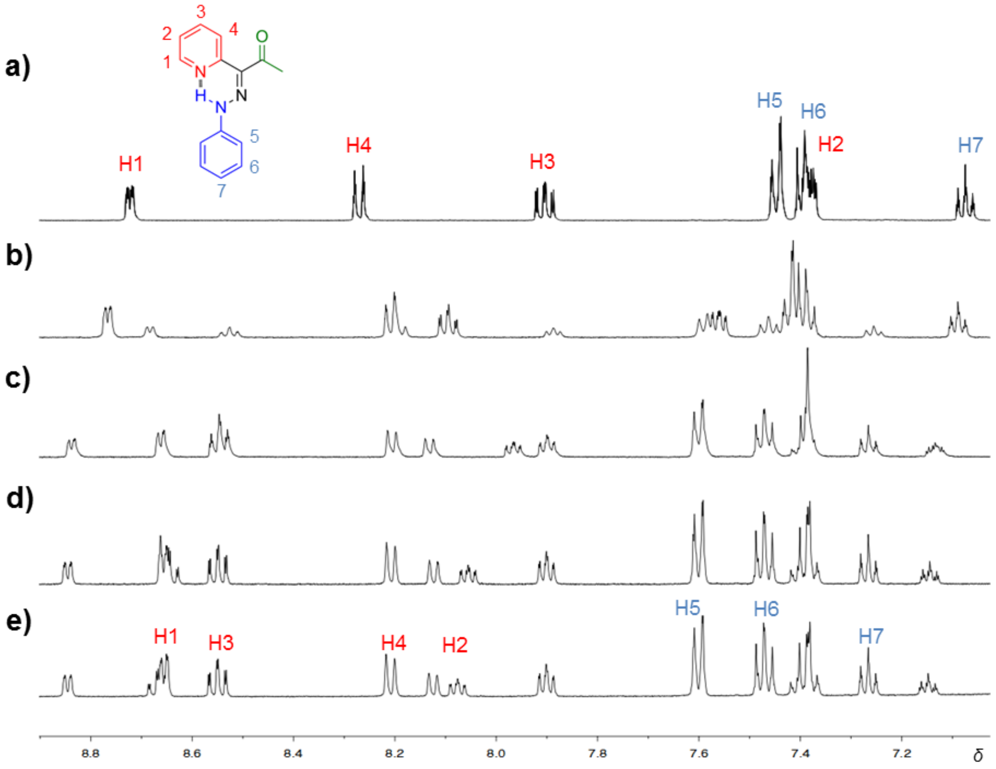

Figure S4: The titration of **PPH-5** with TFA followed by ^{1}H NMR spectroscopy (CD_3CN, 294 K). The **PPH-5** solution (1.0×10^{-2} M) was titrated with 0, 2.4, 10, 20 and 30 equiv of TFA (**a**–**e** respectively).
